# A study on the demand for high-level talent recruitment in tertiary public hospitals in Chongqing based on the Kano model

**DOI:** 10.3389/fpubh.2025.1682732

**Published:** 2025-11-11

**Authors:** Meng Liu, Siyu Wen, Shujie Dai, Quanxin Deng, Jing Li

**Affiliations:** Center for Medical and Social Development Research, School of Public Health, Chongqing Medical University, Chongqing, China

**Keywords:** Kano model, talent recruitment demands, high-level talent, public hospitals, Chongqing

## Abstract

**Objective:**

This study examines the recruitment demands for high-level talent at tertiary public hospitals in Chongqing, providing scientific evidence to assist hospital administrators in formulating optimized strategies for attracting such personnel.

**Method:**

A stratified random sampling method was employed to investigate the recruitment demands for high-level talent in tertiary public hospitals in Chongqing. Quantitative analysis was conducted using the Kano model and Better-Worse matrix analysis.

**Results:**

The Kano analysis identified that among the 20 high-level talent recruitment demands, the majority were categorized as one-dimensional and attractive demands, with only talent incentive schemes, career development opportunities, and performance appraisal systems being must-be demands. The Better-Worse analysis revealed 6 must-be, 5 attractive, 6 one-dimensional, and 3 indifferent demands. Ranked according to the priority of high-level talent recruitment demands, the top five demands are: a scientifically sound and reasonable performance appraisal system; opportunities for professional development; talent incentive measures; receiving respect and care; and generous remuneration packages.

**Conclusion:**

In the recruitment of high-level talents, Must-be demands are the core factor, One-dimensional demands are the paramount priority, and attractive demands serve as supplementary factors. When formulating talent recruitment strategies, hospital administrators should adopt targeted measures to prioritize fulfilling the must-be demands of high-level talents, enhance their one-dimensional demands, and elevate their attractive demands. They must fully consider the importance and priority of different demands, identify the key strengths and weaknesses in talent acquisition, and continuously and dynamically monitor shifts in the demands of high-level talent.

## 1 Introduction

High-level talents generally refer to individuals holding doctoral degrees or senior professional titles, capable of spearheading disciplinary advancement through their extensive expertise and exceptional professional capabilities (1). They serve as both a vital guarantee for driving the high-quality and innovative development of public hospitals (2) and a key factor in enhancing the quality of healthcare services.

The report to the 20th National Congress of the Communist Party of China emphasized the need to uphold the guiding principles of educating for the Party and nurturing talent for the nation. Cultivating high-level talent remains the central theme of talent development and reform. For healthcare institutions, particularly public hospitals, there is an urgent need to strengthen the development of high-level medical talent. The 14th Five-Year Plan for the Development of Chongqing’s Health and Healthcare Sector proposes achieving high-quality development in health and healthcare by 2025. This involves establishing a high-quality, efficient medical and healthcare service system; building a nationally renowned medical city; creating a leading medical hub in western China; and establishing a nationally recognized medical center with considerable international influence (3). The achievement of this goal hinges on the support of high-level talent. However, the scarcity and irreplaceability of such talent (4) make their recruitment particularly challenging.

Against this backdrop, the focus of Chongqing’s healthcare sector has shifted to how to attract high-level talent, strengthen the development of high-level talent teams, and support the high-quality development of public hospitals. The recruitment of high-level talent requires substantial resource investment. However, effectively identifying and addressing the diverse demands of such personnel has become a critical consideration in the talent recruitment strategies of public hospitals. Therefore, this study examines the recruitment demands for high-level talent at tertiary public hospitals in Chongqing from the perspective of high-level talent demand. Utilizing the Kano model, it analyses the core attributes of talent recruitment demands for these hospitals, aiming to categorize these attributes and establish a prioritization framework. This approach enables the formulation of targeted optimization strategies for recruiting high-level talent in public hospitals, with the objective of strengthening the development of high-level talent teams, enhancing overall medical service quality and capacity, and promoting the high-quality development of public hospitals.

The Kano model was first developed by Japanese professor Noriaki Kano, who drew inspiration from the “two-factor theory” to classify and evaluate service factors using a two-dimensional quality model. This model establishes a dual-dimensional cognitive framework of “quality attribute realization-user satisfaction” based on the degree of product quality attribute realization and customer satisfaction (5). The Kano model is not a customer satisfaction model, but rather a qualitative framework for classifying and analyzing customer needs. It serves as an auxiliary tool for satisfaction evaluation, identifying entry points for enhancing customer satisfaction. This model, grounded in the interplay between actual product performance and customers’ subjective experiences, identifies a series of relational attributes within a two-dimensional framework. It aims to categorize and prioritize customer needs (6, 7). In 2003, Spanish scholars Jane and Dominguez were the first to systematically elaborate on the theoretical and methodological application of the Kano model to the healthcare industry (8). In the field of public health services, the Kano model is primarily applied to research on hospital service quality improvement, patient satisfaction enhancement, and healthcare personnel needs analysis. Jorge et al. employed the Kano model to survey 204 migraine patients and 68 neurologists, analyzing the disparity in treatment expectations between patients and physicians during migraine management (9). Li Zhang et al. introduced the Kano model into radiology to stratify the timing demands for receiving MRI reports among clinic staff and physicians (10). Deng Mengzhu employed the Kano model to analyze the demand attributes of patients and medical staff regarding non-medical technical services in outpatient settings, thereby proposing strategies to enhance such services (11). Vassiliadis et al. surveyed patients at a Greek public secondary hospital, employing the KANO model to establish a patient satisfaction dimension analysis. They explored how patient satisfaction with service quality is influenced by new facilities, identifying key factors affecting patient satisfaction and areas for targeted improvement (12). Zhang Yili et al. conducted a Kano survey questionnaire among 1,368 primary-level medical personnel in Guangdong Province. The study identified 24 survey items reflecting healthcare workers’ needs, categorized as follows: 4 essential needs, 9 expected needs, and 11 delight needs. These findings provide a reference basis for refining incentive mechanisms for primary-level medical personnel and enhancing their satisfaction and work motivation (13). It is evident that existing research has yet to employ the Kano model to conduct quantitative analysis and prioritization of talent recruitment demands within public hospitals.

## 2 Research subjects and methods

### 2.1 Subject of study

This study conducted a questionnaire survey from December 2024 to March 2025 targeting high-level talents recruited by tertiary public hospitals in Chongqing. Inclusion criteria were: medical professionals holding a doctoral degree or possessing associate senior professional titles and above, including clinical, research, management, and technical personnel. All participants provided informed consent and voluntarily participated in the questionnaire survey.

### 2.2 Sample size calculation

According to Xiao Shunzhen’s sample size estimation method (14), the calculation is based on 5 to 10 times the number of questionnaire items, accounting for a 10 to 30% sample loss rate. That is, *n* = max(number of items) × (5–10) × (1 + (10–30%)). The calculated total sample size n ranges from [110–260], with a minimum sample size of 110.

### 2.3 Sampling method

First, the 38 administrative districts and counties of Chongqing Municipality are categorized into four tiers based on their economic development levels (per capita GDP): the Central Urban Districts, the Main Urban New Districts, the Town Cluster in the Northeast Chongqing Three Gorges Reservoir Area, and the Town Cluster in the Southeast Chongqing Wuling Mountain Area. As of May 2024, Chongqing Municipality has a total of 50 Class A Grade III public hospitals, including 24 in the central urban area, 19 in the new urban districts, 6 in northeastern Chongqing, and 1 in southeastern Chongqing. A random sample of 10 tertiary public hospitals was selected as the research sample source, drawn proportionally at 1/5: 5 tertiary public hospitals from the central urban area, 3 from the main urban new district, and 1 each from northeastern and southeastern Chongqing. A voluntary online survey was conducted from December 2024 to March 2025 targeting high-level talents recruited by tertiary public hospitals in Chongqing. A total of 135 questionnaires were collected. Following preliminary screening via questionnaire analysis software (QuestionStar) and manual verification, two identical responses were excluded, yielding 133 valid questionnaires. This represents an effective response rate of 98.5%.

### 2.4 Questionnaire design

#### 2.4.1 Designing the survey questionnaire

This study draws upon the dual-factor theory (15) and amenities theory (16) as its theoretical foundation. It references research by scholars such as Lin Z (17) and Gu W (18), integrates relevant policies for attracting high-level talent in Chongqing, and follows the design principles of the Kano model. Consequently, a customized questionnaire was developed to investigate the recruitment demands for high-level talent in Chongqing’s top-tier public hospitals. The questionnaire encompasses four dimensions: livelihood security and welfare, work value and interpersonal experiences, talent development and comprehensive capabilities, and city environments conducive to both work and residence. It includes 20 survey items on demands, as shown in Table 1.

**Table 1:** Specific items in the questionnaire on the recruitment of high-level talent in public hospitals.

Dimension	Specific entries
A. Living Allowances and Benefits	A1. Compensation Package (33)
	A2. Housing allowance or housing subsidy (34)
	A3. Spousal Employment (18)
	A4. Children’s School Enrollment (18)
B. Work Value and Interpersonal Experience	B1. Job satisfaction (33)
	B2. Challenging work (33)
	B3. Interpersonal Relationships (33)
	B4. Respect and care
C. Talent Development and Comprehensive Strength	C1. Career Development Opportunities (33)
	C2. Learning and Growth Platform and Opportunities (34)
	C3. Talent Incentive Measures (17)
	C4. Performance Appraisal System (33)
	C5. Level of Disciplinary Development (17)
	C6. Research Capabilities and Support (17)
	C7. Work Environment (33)
D. A city with a business-friendly and livable environment	D1. Talent Recruitment Policy (17)
	D2. Level of economic development (35)
	D3. Urban Public Services (35)
	D4. Natural Ecological Environment (35)
	D5. Urban Infrastructure Development (35)

#### 2.4.2 Questionnaire content

The questionnaire is divided into the following two parts. ① General information survey form, including gender, age, marital status, educational background, job category, professional title, years of work experience, annual income, etc. ② Survey on the demands for attracting high-level talent based on the Kano model, with a total of 20 survey items. Each item is asked from both positive and negative perspectives to measure the attitude of high-level talent toward the various items in the talent recruitment process at top-tier public hospitals, both when the items are present and when they are absent. Taking “compensation and benefits” as an example, the question is posed as follows: “If the talent recruitment program offers generous compensation and benefits, how do you feel?,” “If the talent recruitment does not offer generous compensation and benefits, how do you feel? “The response options are: “Dislike,” “Can tolerate,” “Indifferent,” “Should be expected” and “Like,” with five options in total.

#### 2.4.3 Preliminary survey

A preliminary survey was conducted on 50 high-level talents selected at random. The Cronbach’s *α* coefficients for the forward and reverse questionnaires were 0.930 and 0.980, respectively, indicating excellent reliability of the questionnaires. The KMO values for the positive and negative questionnaires were 0.743 and 0.902, respectively, with Bartlett’s sphericity test *p*-values both below 0.001. This indicates correlations among questionnaire items and good structural validity. Validity testing of the 20 positive–negative demand survey indicators using principal component factor analysis yielded four primary factors, collectively explaining 77% of the variance. Among these, Factor 1 includes spouse employment, children’s schooling, relocation allowance or housing subsidy, and compensation packages; Factor 2 encompasses job satisfaction, work challenges, interpersonal relationships, respect, and care; Factor 3 covers career advancement opportunities, learning and growth platforms and opportunities, talent incentive measures, performance appraisal systems, disciplinary development levels, research capabilities and support, and work environment; Factor 4 comprises talent recruitment policies, urban public services, economic development levels, natural ecological environment, and urban infrastructure development.

### 2.5 Research methods

#### 2.5.1 Kano attribute classification

**Figure 1:**
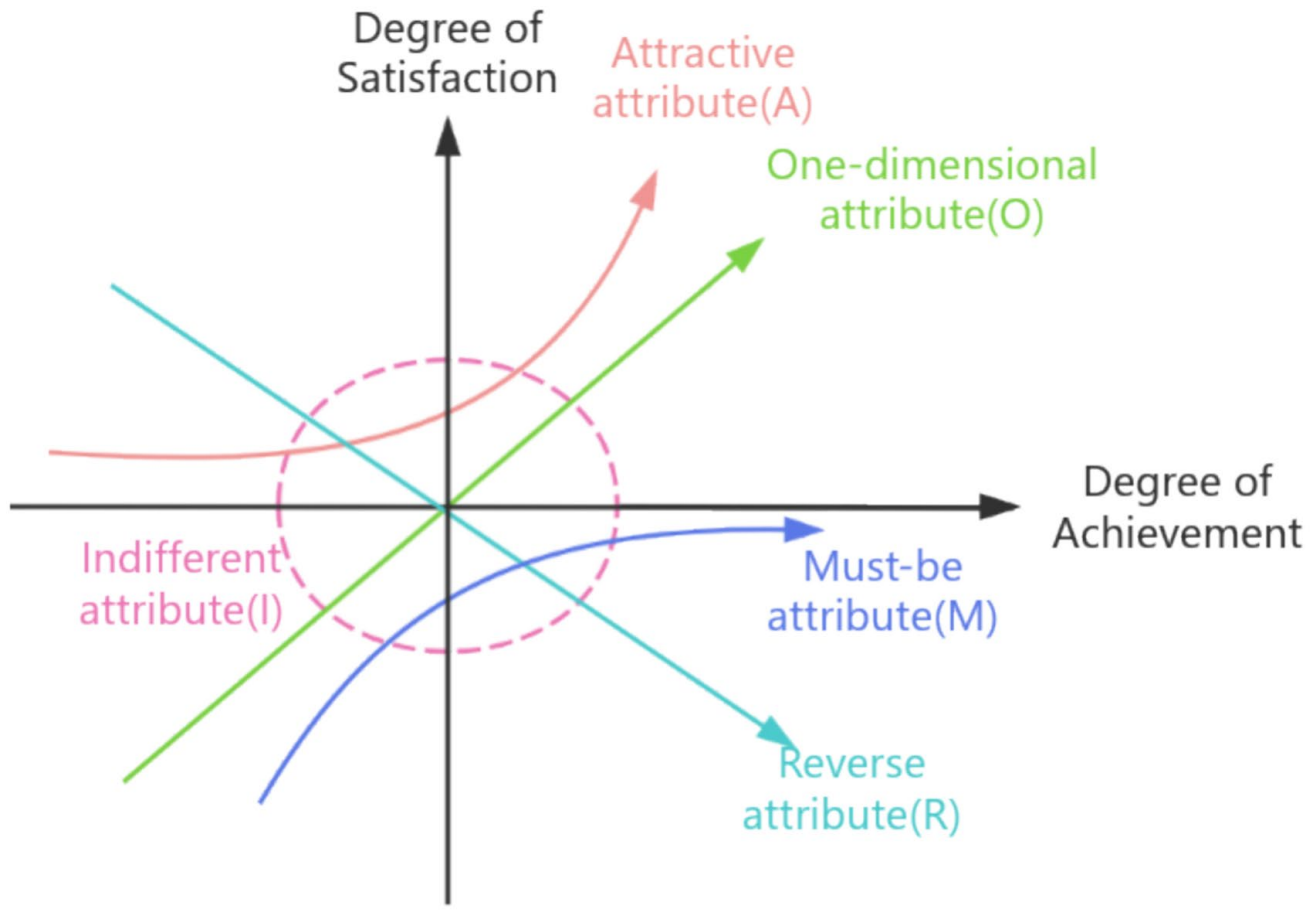
The five attributes of demand in the Kano model.

Following the traditional classification method for Kano model attributes (see Table 2), the frequency statistics for each item across different attribute categories were calculated. The attribute with the highest proportion represents the demand attribute category for that item. This study categorizes the attribute categories for the recruitment of high-level talent at tertiary public hospitals into five types: must-be demands (M), one-dimensional demands (O), attractive demands (A), indifferent demands (I), and reverse demands (R) (19). As shown in Figure 1.

**Table 2:** Kano model satisfaction two-dimensional model classification.

Positive question	Negative question				
	Like	Should be expected	Indifferent	Can tolerate	Dislike
Like	Q	A	A	A	O
Should be expected	R	I	I	I	M
Indifferent	R	I	I	I	M
Can tolerate	R	I	I	I	M
Dislike	R	R	R	R	Q

A stands for attractive quality, O stands for one-dimensional quality, M stands for must-be quality, I stands for indifferent quality, R stands for reverse quality, and Q stands for questionable quality.

#### 2.5.2 Better-worse coefficient calculation

Based on the Kano attribute classification results, calculate the Satisfaction Influence (SI) - Dissatisfaction Influence (DSI) coefficient, also known as the Better-Worse coefficient. Better values range between [0,1]; the larger the value or the closer it is to 1, the greater the satisfaction increase for high-level talent when this demand is met. Worse values range between [−1,0]; the larger the absolute value or the closer it is to 1, the faster the satisfaction decline for high-level talent when this demand is not met. The specific formula is as follows (20): Better = (A + O) / (A + O + M + I), i.e., (Attractive Factor + One-dimensional Factor) / (Attractive Factor + One-dimensional Factor + Must-be Factor + Indifferent Factor); Worse = −1 × (O + M) / (A + O + M + I), i.e., −1 × (One-dimensional Factor +Must-be Factor) /(Attractive Factor + One-dimensional Factor + Must-be Factor + Indifferent Factor).

#### 2.5.3 Kano model matrix analysis

**Figure 2:**
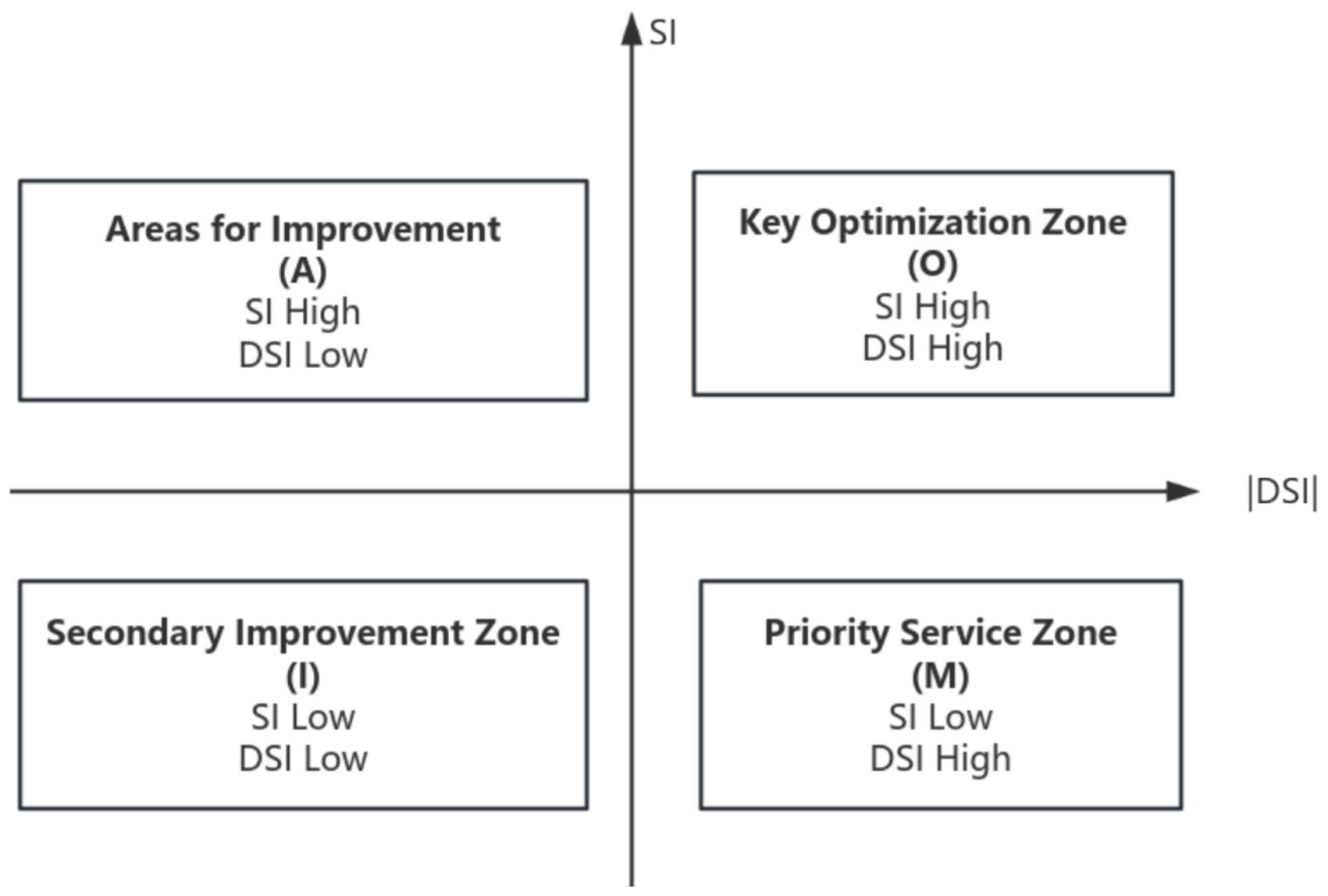
Better-Worse matrix analysis.

Based on the Better-Worse coefficients for each demand, plot a two-dimensional Better-Worse matrix diagram (as shown in Figure 2). The absolute value of the Worse coefficient serves as the x-axis, while the Better coefficient forms the y-axis. The origin is defined by the average of the absolute Worse value and the Better value, dividing the plane into four quadrants. The first quadrant represents the priority optimization zone (one-dimensional demand). Both the Better-Worse coefficients in this quadrant exhibit high absolute values, indicating that if all recruitment demands in this area are met, the satisfaction of high-level talent in public hospitals will rapidly increase; conversely, it will rapidly decline. The second quadrant represents the improvement zone (attractive demand). This quadrant exhibits a high Better value but a low absolute Worse value, indicating that meeting talent acquisition demands in this area will exceed talent expectations and significantly boost satisfaction levels. Failure to address these demands, however, will not negatively impact talent satisfaction. The third quadrant represents the secondary improvement area (indifferent demand), where both the Better-Worse coefficients are relatively low, indicating no impact on satisfaction levels among high-level talent. The fourth quadrant represents the priority fulfillment zone (must-be demand). This quadrant exhibits a lower Better value but a higher absolute Worse value, indicating that fulfilling recruitment demands in this area will not enhance talent satisfaction. However, failing to meet these demands will cause talent satisfaction to decline significantly, demonstrating that these are the most fundamental and indispensable demands for high-level talent (20).

### 2.6 Statistical methods

A database was established using Excel 2010, with statistical analysis and processing conducted via both Excel 2010 and SPSS 26.0. Descriptive analysis of general data employed relative indicators such as composition ratios. Through the Kano model attribute classification method, the attribute with the highest frequency proportion among M, O, A, I, R, and Q for each item was designated as its final attribute. The Better-Worse coefficients and their average for each demand were automatically calculated by Excel 2010, while the Better-Worse two-dimensional matrix analysis diagram was generated by SPSS.

## 3 Results

### 3.1 General characteristics of the survey participants

Among the 133 participants in this survey, 77 were male (57.89%) and 56 were female (42.11%); Age distribution: 3 participants under 30 (2.26%), 58 aged 31–40 (43.61%), 58 aged 41–50 (43.61%), and 14 aged 51 and above (10.53%); Marital status distribution: 122 participants were married (91.73%); Educational background: Doctoral degree holders: 126 (94.74%); Master’s degree holders: 5 (3.76%); Bachelor’s degree holders: 2 (1.5%); Job categories of recruited talent: Traditional Chinese Medicine practitioners: 53 (39.85%); Research: 59 (44.36%); Medical technology: 13 (9.77%); Pharmacists: 4 (3.01%); management (3 individuals, 2.26%), and other roles (1 individual, 0.75%); The most common professional titles were senior and associate senior levels, comprising 73 individuals (54.89%) and 46 individuals (34.59%), respectively; Work experience predominantly fell into the 6–10 years and over 10 years categories, with 65 individuals (48.87%) and 49 individuals (36.84%), respectively.

### 3.2 Traditional classification results of high-level talent recruitment demands in tertiary public hospitals

Based on the Kano model, a two-dimensional attribution analysis was conducted on the demand for high-level talent, with the attribute with the highest frequency being identified as the demand attribute for this project. The results showed that: among the 20 items of talent recruitment demands in tertiary public hospitals, 3 were must-be demands (accounting for 15%), 9 were one-dimensional demands (accounting for 45%), and 8 were attractive demands (accounting for 40%). There were no reverse demands, indifference demands, or questionable results (see Table 3).

**Table 3:** High-level talent recruitment demands of tertiary public hospitals in Chongqing (*n* = 133).

Items	M	O	A	I	R	Q	Demand attributes	Better(SI)	Worse(DSI)
Talent incentive measures	**57**	40	30	6	0	0	M	0.526	−0.729
Career development opportunities	**61**	57	11	4	0	0	M	0.511	**−0.887**
Performance appraisal system	**82**	33	13	5	0	0	M	0.346	**−0.865**
Interpersonal relationships	12	**90**	26	5	0	0	O	**0.872**	**−0.767**
Job satisfaction	20	**83**	26	4	0	0	O	**0.819**	**−0.774**
Work environment	13	**64**	44	12	0	0	O	0.812	−0.579
Learning and growth platform	29	**70**	32	2	0	0	O	0.767	−0.744
Talent introduction policy	16	**56**	43	18	0	0	O	0.744	−0.541
Housing allowance	24	**57**	41	11	0	0	O	0.734	−0.609
Urban infrastructure construction	31	**50**	45	7	0	0	O	0.714	−0.609
Respect and care	31	**78**	16	8	0	0	O	0.707	**−0.819**
Compensation package	30	**62**	27	14	0	0	O	0.670	−0.692
Natural environment	3	34	**86**	10	0	0	A	**0.902**	−0.278
Level of economic development	2	18	**101**	12	0	0	A	**0.895**	−0.150
Urban public services	13	34	**78**	8	0	0	A	**0.842**	−0.353
Level of academic development	15	28	**80**	9	0	1	A	0.818	−0.326
Research capabilities and support	18	44	**60**	11	0	0	A	0.782	−0.466
Children’s enrollment in school	3	21	**72**	37	0	0	A	0.699	−0.181
Spouse employment	1	19	**68**	45	0	0	A	0.654	−0.150
Work Challenge	3	18	**57**	55	0	0	A	0.564	−0.158

The bolded sections, denote the final attributes of the respective requirements and their key coefficients.

The aforementioned traditional Kano analysis classification reveals that attractive demands and one-dimensional demands constitute 85% of the demand attributes for high-level talent. These demands are what the vast majority of high-level talent are eager to obtain or expect to receive. They include interpersonal relationships, respect and care, a sense of achievement and challenge in work, the working environment, learning and growth platforms and opportunities, settlement allowances or housing subsidies, remuneration packages, academic discipline development standards, research support, spouse and child settlement arrangements, as well as the city’s talent recruitment policies, economic development levels, natural ecological environments, infrastructure development, and urban public services. These encompass crucial aspects of public hospital development, the personal advancement of high-level talent, and the city’s inherent appeal. Additionally, three must-be demands are identified: the provision of talent incentive measures, the availability of career development pathways and promotion opportunities, and a scientifically sound and reasonable performance appraisal system.

### 3.3 Better-worse coefficient and two-dimensional matrix analysis of talent recruitment demands for high-level talent in tertiary public hospitals

The results of the Better-Worse coefficient calculation show that the Better range is (0.346, 0.902), and the Worse range is (−0.887, −0.150), see Table 3. Among these, the top five demands ranked by satisfaction impact are: the city has a suitable natural ecological environment (0.902), the city has a high level of economic development (0.895), good interpersonal relationships in the workplace (0.872), the city has high-quality and efficient public services (0.842), and a sense of accomplishment from work (0.819),indicates that this demand has a significant impact on enhancing the satisfaction levels of high-level talent. The top five demands ranked by dissatisfaction impact are: Having career development opportunities and promotion prospects (−0.887), Having a scientific and reasonable performance appraisal system (−0.865), Receiving respect and care at work (−0.819), Having a sense of accomplishment from work (−0.774), and Having good interpersonal relationships at work (−0.767). This indicates that the demand has a significant impact on the decline in satisfaction among high-level talent.

**Figure 3:**
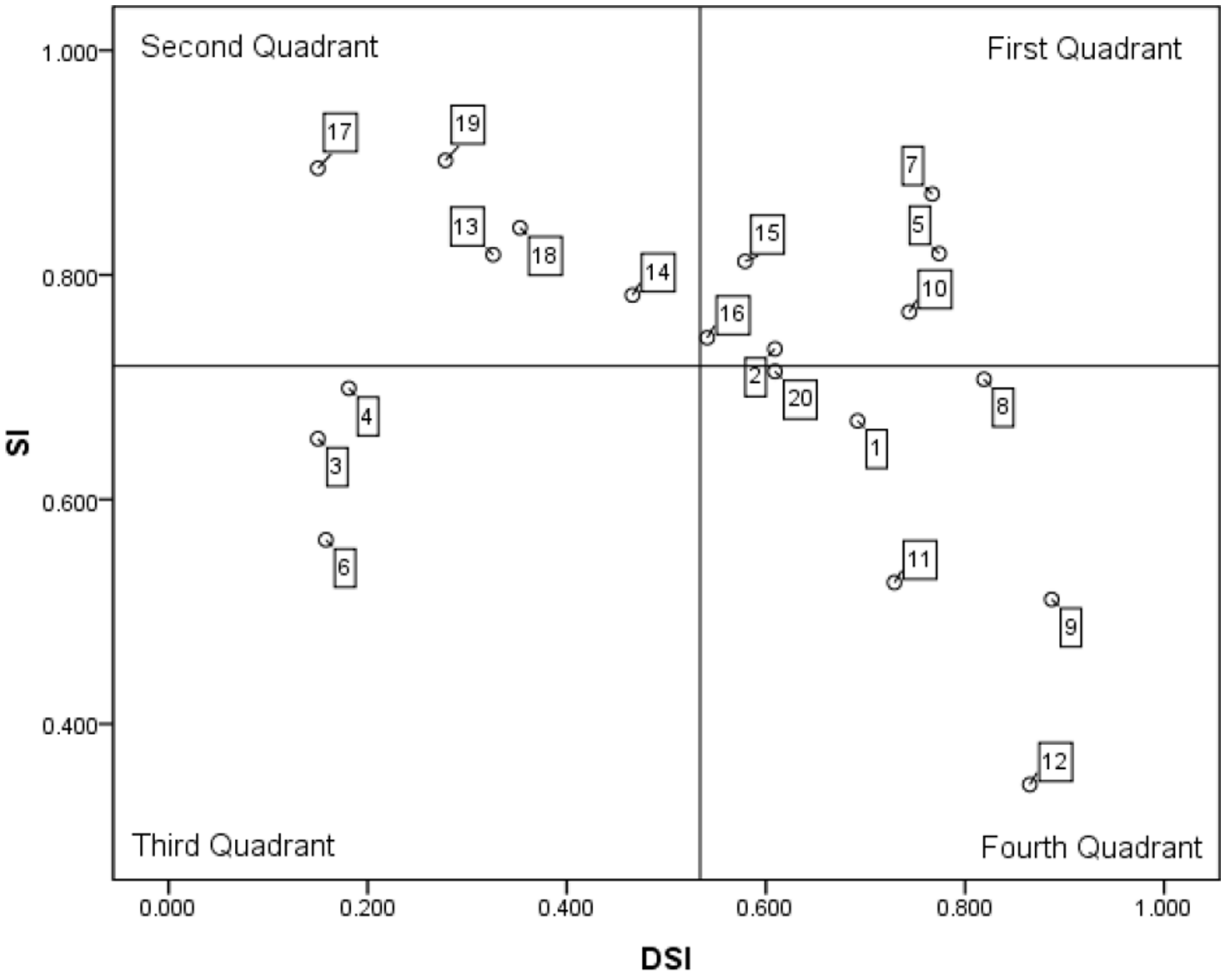
Better-worse two-dimensional matrix of high-level talent recruitment demands.

Plotting the absolute value of Worse on the x-axis and the Better value on the y-axis, a four-quadrant diagram is constructed based on the Better-Worse indices from Table 3. This is achieved by dividing the plane using the average of the two indices (0.72, |−0.53|) as the origin, as illustrated in Figure 3.

### 3.4 Priority ranking analysis of high-level talent recruitment demands

Due to certain limitations inherent in the traditional Kano analysis method, which overlooks the influence of other attribute dimensions, the attribute categories for each demand item exhibit no discernible differentiation. Therefore, this study adopts the statistical results of the Better-Worse Index Quadrant Analysis method as the basis. By integrating the traditional Kano model’s ranking of attribute importance: Must-be attributes > One-dimensional attributes >Attractive attributes > Indifferent attributes (21), and excluding three indifferent demands with negligible impact on satisfaction (spouse’s employment, children’s schooling, and job challenge), we identify 17 demands prioritized by high-level talent. Concurrently, applying the Better-Worse coefficient analysis principle—where demands farther from the origin in the quadrants exert greater influence on high-level talent satisfaction—priority rankings were assigned to each demand based on importance (See Table 4).

**Table 4:** Priority ranking of high-level talent recruitment demands in public hospitals.

Talent recruitment demands	Demand attribute classification	Priority ranking
12. Performance Appraisal System	M	1
9. Career development opportunities	M	2
11. Talent Incentive Measures	M	3
8. Respect and care	M	4
1. Compensation package	M	5
20. Urban infrastructure construction	M	6
7. Interpersonal Relationships	O	7
5. Job satisfaction	O	8
10. Learning and Growth Platform	O	9
15. Work Environment	O	10
2. Housing allowance	O	11
16. Talent introduction policy	O	12
17. Level of economic development	A	13
19. Natural environment	A	14
13. Level of academic development	A	15
18. Urban public services	A	16
14. Research capabilities and support	A	17

## 4 Discussion

### 4.1 Accurately identify recruitment demands for high-level talent and refine demand factors based on attributes

According to the research hypothesis of the Kano model, satisfying attractive demands has the most significant effect on improving high-level talent satisfaction, while fulfilling must-be and one-dimensional demands is more effective in preventing dissatisfaction among high-level talent. When formulating optimization strategies for recruiting high-level talent, hospital administrators should first clarify whether the optimization goal is to enhance high-level talent satisfaction or reduce dissatisfaction among high-level talent (11).

#### 4.1.1 Must-be demands are the most critical factor influencing the satisfaction of high-level talent

Neglecting these demands can lead to a significant decline in satisfaction, prompting hospital administrators to prioritize addressing such demands for high-level personnel. The six must-be demands, prioritized in order of development sequence, are: performance appraisal systems; career development prospects and promotion opportunities; talent incentive measures; respect and care; remuneration packages; and urban infrastructure development. Among these, “career development opportunities and promotion prospects” ranks first in the dissatisfaction coefficient affecting high-level talent, demonstrating that this demand is highly valued by them. These findings align with the research of Bailey (22) and Garcia-Rodriguez (23), which suggests that the realization of personal career values and career trajectories play a crucial role in talent mobility decisions. High-level talents place great emphasis on personal development. This stems from their status as knowledge workers possessing strong technical expertise, which makes them highly focused on personal growth, creative, and mobile (24). For high-level talent, work serves as a means to demonstrate their capabilities and realize their aspirations. They are more inclined toward achieving significant breakthroughs in technology and theory, thereby fulfilling their self-worth (25). Generous remuneration packages are a significant factor in attracting high-level talent (18). Hagander conducted empirical research on surgeon migration between developing nations and the United States, finding that compensation and family considerations are key drivers of talent repatriation (26). High-level talents also prioritize respect in the workplace. Respect and care, as vital sources of psychological income for such talent, significantly influence their decision to relocate (27).

#### 4.1.2 One-dimensional demands are linear factors influencing the satisfaction of high-level talent

The six demands falling under one-dimensional attributes, prioritized in order of development sequence, are: interpersonal relationships; job satisfaction; learning and growth platforms and opportunities; working environment; relocation allowance or housing subsidy; and talent recruitment policies. These constitute the primary demands for attracting high-level talent. Continually optimizing these can significantly enhance their satisfaction levels, and resources should be mobilized to meet these one-dimensional demands to a high standard. In particular, both the Better values and absolute Worse values for ‘interpersonal relationships’, ‘sense of achievement at work’, and ‘learning and growth platforms and opportunities’ exceed 0.7, indicating that these one-dimensional demands should be prioritized for fulfillment. This finding aligns with Tlaiss’s research (28), which also suggests that a positive work environment and good relationships among colleagues enhance talent satisfaction and serve as crucial measures for retaining top talent. For talent, relocation allowances or housing subsidies can reduce living costs and alleviate the pressure of securing accommodation; they also serve as essential safeguards for psychological security. Consequently, housing benefits (such as relocation allowances, housing subsidies, property purchase incentives, and talent apartments) significantly influence talent recruitment efforts (29). In an era of rapid knowledge renewal, high-level talents seek continuous enhancement of their specialized skills and knowledge base. Hospitals should provide platforms for learning and growth through academic exchange activities, professional training programmers, and research project support. This enables talent to engage with cutting-edge industry information and advanced technologies, facilitating breakthroughs in their personal career development.

#### 4.1.3 Attractive demands serve as enhancing factors influencing the satisfaction of high-level talent

The five demands pertaining to the attractive attribute, prioritized in order of development sequence, are as follows: Urban economic development level, Urban natural ecological environment, Hospitals possessing advanced disciplinary development standards, Efficient urban public services, Strong research capabilities and support. This research finding aligns with Ullman’s (16) conclusion that “urban natural environment and quality of life are key determinants of talent mobility.” High-level talent places greater emphasis on quality of life and prioritizes the comfort of urban living. Therefore, providing high-level talent with a comfortable natural ecological environment, efficient urban public services, and a high level of economic development can effectively enhance a city’s attractiveness to talent (30). Compared to one-dimensional and must-be attributes, the absolute values for Worse are lower and Better values are higher for attractive attributes. This indicates that if the attractive demands can be met, the satisfaction levels of high-level talent will increase significantly; however, if this demand is not met, it has no significant impact on their dissatisfaction. Therefore, having met must-be and one-dimensional demands, Chongqing’s public hospitals and their governing bodies should actively address and invest in such demands to enhance their competitiveness in the healthcare talent market and attract more high-level talents.

### 4.2 Identify key talent acquisition steps to prioritize resource allocation

Tertiary public hospitals should allocate limited resources to the most appropriate areas. When resources are severely constrained, eliminate indifferent demands to prevent waste. Based on the analysis results of the Better-Worse two-dimensional matrix, clearly identify priority demands, key optimization demands, demands awaiting improvement, and secondary improvement demands. This enables precise alignment of resources with demands, avoids mismatches between supply and demand, and achieves twice the result with half the effort. Aligning with the strategic positioning of tertiary public hospitals and the realities of high-level talent, the focus is placed on demands that most significantly impact satisfaction and dissatisfaction. Tailored, personalized improvement strategies are then developed for different types of requirements, thereby achieving the goal of continuously enhancing the satisfaction of high-level talent.

The results of the Better-Worse Matrix analysis indicate that, the talent recruitment demands for public hospitals situated in the area for improvement (second quadrant) comprise five attractive demands. Three of these fall under the dimension of the city’s favorable working and living environment, while two relate to the hospital’s comprehensive capabilities. Policy makers should enhance city promotion and image building, highlighting the city’s strengths. For instance, Chongqing should showcase its recent economic achievements, habitable natural environment, and efficient public services, integrating these with talent recruitment policies to form an attractive ‘comprehensive package’. Concurrently, investments in hospital discipline development should be increased, advanced medical equipment and technologies introduced, and collaborations with renowned domestic and international medical institutions strengthened. High-level research and innovation platforms—such as national laboratories and clinical research centers—should be established to provide talent with favorable research environments and innovative conditions.

The talent recruitment demands for public hospitals situated in the priority fulfillment zone (fourth quadrant) comprise six must-be demands. Regarding performance appraisal, the irrationality of hospital assessment systems and standards directly undermines staff motivation and constrains talent utilization, creating a vicious cycle of ‘high expectations and low efficacy’ (31). Consequently, healthcare administrators and hospital management must begin with top-level design of the assessment framework: establishing a personalized evaluation system based on categorized talent management, with tailored metrics for each individual’s role and level. Regarding career progression, hospitals should respect natural growth patterns by designing bespoke advancement pathways for different talent types, avoiding one-size-fits-all promotion criteria to provide high-level professionals with clear developmental trajectories. Regarding talent incentives, hospitals should adopt multi-tiered, comprehensive motivational measures. Prioritizing the career development of high-level talent, hospitals can implement achievement-based incentives. This may involve conferring honor and titles, or providing workplace conveniences to enhance their sense of accomplishment (32). Such approaches also encourage them to center their professional development around the hospital, thereby strengthening their sense of honor and responsibility.

### 4.3 Dynamically monitor changes in talent demand attributes and continuously refine demand factors

Professor Kano’s research series revealed that as time and environments evolve, the demand types defined by the Kano model exhibit progressive development and follow distinct lifecycle. Driven by time and competition, the demands of high-level talent evolve: Indifferent demands→ Attractive demands→One-dimensional demands→Must-be demands. Therefore, public hospitals should dynamically monitor high-level talent needs, regularly conduct Kano model analyses, investigate and understand potential demands, grasp the cycles of demand transformation, and continuously refine the demand elements for high-level talent. This approach will steadily enhance their work motivation and satisfaction.

### 4.4 Limitation

Firstly, cross-sectional data can only reflect the static characteristics of high-level talent recruitment demands in public hospitals at a specific point in time, failing to capture the dynamic evolution of such demands. Secondly, self-reporting scales may be subject to social desirability bias and subjective judgment bias, thereby compromising the accuracy of demand analysis. Finally, the study’s regional scope is limited. Constrained by finite human, material, and financial resources, this research is confined to tertiary public hospitals in Chongqing Municipality. Consequently, its conclusions may not be generalizable nationwide. Future research could expand the survey area to encompass additional provinces (municipalities), thereby deepening the exploration of public hospitals’ demand for high-level talent recruitment. This would provide a foundation for hospital talent recruitment policies and practical implementation.

## 5 Conclusion

This study examines the recruitment requirements for high-level talent at tertiary-level public hospitals in Chongqing. By applying the Kano model and Better-Worse index analysis, it distinguishes and ranks the demand attributes of each recruitment criterion. This provides a quantitative analytical framework and an intuitive matrix analysis diagram for accurately identifying the demands of high-level talent. This holds significant importance for hospital administrators in refining talent recruitment strategies, enhancing the quality of hospital talent management, and improving staff satisfaction with human resources initiatives. Hospitals should strive to fulfill the must-be demands of personnel, meet their one-dimensional demands to a high standard, and endeavor to enhance their attractive demands. Concurrently, a dynamic monitoring system for personnel demands should be established to refine the elements of personnel demands in response to shifts in demand.

## Data Availability

The original contributions presented in the study are included in the article, further inquiries can be directed to the corresponding author.
